# Psychometric Validation and Reference Norms for the European Spanish Developmental Coordination Disorder Questionnaire: DCDQ-ES

**DOI:** 10.3390/ijerph17072425

**Published:** 2020-04-02

**Authors:** Rebeca Montes-Montes, Laura Delgado-Lobete, Javier Pereira, Sergio Santos-del-Riego, Thais Pousada

**Affiliations:** 1 TALIONIS Research Group, Research Centre of the Galician University System. Centre for Information and Communications Technology Research (CITIC), Universidade da Coruña, 15008 A Coruña, Spain; rebeca.montes@udc.es (R.M.-M.); javier.pereira@udc.es (J.P.); thais.pousada.garcia@udc.es (T.P.); 2 Health Integration and Promotion Research Unit (INTEGRA SAÚDE), Faculty of Health Sciences, University of A Coruña, 15011 A Coruña, Spain; sergio.santos.delriego@udc.es

**Keywords:** developmental coordination disorder, validity, reliability, screening, parental questionnaire, developmental coordination disorder questionnaire

## Abstract

The Developmental Coordination Disorder Questionnaire (DCDQ) is a widely used and well-validated tool that contributes to the diagnosis of Developmental Coordination Disorder (DCD). The aim of this study was to further analyze the psychometric properties of the European Spanish cross-culturally adapted version of the Developmental Coordination Disorder Questionnaire (DCDQ-ES) in a sample of Spanish children aged 6–11 years and to establish reference norms with respect to age groups. Parents of 540 typically developing children completed the DCDQ-ES. A second sample of 30 children with probable DCD (pDCD) was used to test its discriminant validity. Confirmatory factor analysis supported the original three-factor structure and the internal consistency was excellent (Cronbach’s α = 0.907). Significant differences between age groups were found. The pDCD group scored significantly lower than the reference sample in the three subscales and DCDQ-ES total score (*p* < 0.001; AUC = 0.872). The DCDQ-ES is a reliable and valid tool for screening motor coordination difficulties in Spanish children and for identifying children with probable DCD. The findings of this research suggest that context-specific cut-off scores should be systematically utilized when using cross-cultural adaptations of the DCDQ. Age-specific cut-off scores for Spanish children are provided.

## 1. Introduction

It is estimated that Developmental Coordination Disorder (DCD) affects approximately 5%–10% of school-aged children, making it the most prevalent neurodevelopmental disorder in childhood [1,2,3]. Children with DCD present motor coordination difficulties that significantly and persistently limit their daily functioning. As established by the fifth edition of the Diagnostic and Statistical Manual of Mental Disorders (DSM-5), children with DCD must show significantly poorer motor coordination performance than expected from the child’s chronological age and opportunity for skill learning and use (criterion A) that significantly and persistently interferes with typical activities of daily living (criterion B), where onset occurs in the early developmental period (criterion C) and that cannot be better explained by a neurological condition affecting movement (criterion D) [2].

Children with DCD often struggle with associated developmental problems such as physical problems, sensory processing issues and psychosocial and anxiety difficulties in childhood and adolescence [4,5,6,7,8,9,10,11], and their daily participation in activities is significantly limited in comparison to their typically developing peers [12,13]. In addition, there is a high co-occurrence between DCD and Attention Deficit Hyperactivity Disorder (ADHD) and Autism Spectrum Disorders (ASD) [3,14]. 

The impact of DCD on daily functioning and participation has been widely reported. Findings from several studies, including a systematic review of 44 articles conducted by Magalhães et al. [12], show that children with DCD have serious activity and participation issues in both productive, leisure and self-care activities, especially in dressing, eating, toileting, sport and outdoor play participation and school-related activities [1,12,13,14,15,16,17]. In addition, the impact of DCD on everyday performance usually persists during adolescence and adulthood [8,18].

As DCD is a chronic condition with lifelong consequences, it is necessary to identify DCD as early as possible in order to prevent further limitations and promote specific intervention [3,19,20]. 

While motor coordination test batteries such as Movement Assessment Battery for Children-2 are recommended to assess criterion A, their utilization is usually too time- and resource-consuming for early identification or screening. Therefore, questionnaires aimed at parents have been developed as feasible alternatives to identify children at risk of DCD and to assess criterion B in large groups of children [3]. Screening methods to quickly identify children with motor difficulties in Spain are highly needed, as most of the Spanish children with probable DCD go underdiagnosed in Pediatric Primary Care [21,22].

There are several parent and teacher questionnaires available aimed to operationalize criterion B for DCD diagnosis, such as the Movement Assessment Battery for Children-2 checklist, the Children Activity Scales (both parent and teacher versions) or the DCDDaily-Q [3]. The Developmental Coordination Disorder Questionnaire (DCDQ) [23] is the most used measure to identify children with probable DCD [2,3]. 

The DCDQ is a three-dimensional, well-validated and easily accessible measure that was developed in Canada and is aimed at assessing motor performance during daily activities in children aged 5–15 years. In order to use it outside Canada, the DCDQ has been successfully cross-culturally adapted to several languages and countries, including Canadian and European French, German, Brazilian, Italian, Polish, Indi and Latin American Spanish [24,25,26,27,28,29,30,31]. 

The DCDDaily-Q [32] is a newly developed parent questionnaire aimed to comprehensively evaluate motor performance in activities of daily living, including self-care and fine motor and gross motor activities. Although both the DCDQ and the DCDDaily-Q evaluate daily motor performance via parental assessment, the type of activities that are evaluated and the assessments parents are required to make differ between both measures [33].

A European Spanish version of the DCDQ (DCDQ-ES) has been recently translated, cross-culturally adapted and preliminarily validated in Spanish children, but further psychometric validation is needed [34]. Developing custom cut-off points for the Spanish population is also required and recommended, as previous studies have demonstrated that the original proposed cut-off points of the DCDQ may not adjust to South European children [35]. 

The aims of this study were (1) to test the psychometric properties of the DCDQ-ES and (2) to develop country-adjusted reference norms and cut-off points for Spanish children.

## 2. Materials and Methods

### 2.1. Participants, Procedures and Research Ethics

As children with DCD are usually identified at school-age, two samples of children aged 6 to 11 years were included in the study: a normative group (*n* = 540, 50.0% boys, mean age = 8.5, SD = 1.7) and a probable DCD group (pDCD) (*n* = 30, 66.7% boys, mean age = 7.9, SD = 1.2) (Table A1). All children in the pDCD group were identified as having probable DCD using the 95th percentile cut-off score on the Spanish version of the DCDDaily-Q (mean score = 46.9, SD = 7.8) [33]. 

The DCDDaily-Q is a parent questionnaire aimed to operationalize criterion B of the diagnosis of DCD [32]. This measure has demonstrated excellent psychometric properties and capacity to identify children with DCD (Cronbach alpha = 0.85; sensitivity = 88%; specificity = 92%) [32]. All children in the pDCD group had been referred to two rehabilitation centers in Spain for motor performance issues, and some of them had a previous medical diagnosis of a co-occurring neurodevelopmental condition (ADHD = 33.3%, ASD = 13.3%, no co-occurring disorder = 53.3%). None of the children in the pDCD group were receiving specific treatment for DCD.

Participants in the normative group were randomly selected from a previously recruited larger sample that came from fourteen randomly selected mainstream elementary schools located in five locations in northwest, north and center of Spain (northwest = 78.1%, north = 20.2%, center = 1.7%) [21,33]. Most of the children (60.6%) came from a family with high/university education level (i.e., at least one parent held a college degree)**.** Children with a parent-reported diagnosis of a developmental disorder were excluded from this group.

A third group that included children in the normative sample was created to serve as a control group for discriminant validity analysis in order to control for age and sex distribution. Children in the control group were randomly selected from the normative group using age- and sex-stratified sampling to match for exact age and sex with the pDCD group. As the pDCD sample size was small (*n* = 30), a 1:2 ratio was used for the control group (*n* = 60) to increase the statistical power of the analyses [36,37].

This study was approved by the Autonomic Research Ethics of Galicia Committee (code 2017-167). The DCDQ-ES was sent to the parents of the participants between June 2017 and December 2019 via school or rehabilitation center intermediation, so the parents could complete the DCDQ-ES at home. 

Parents also received an informative letter about the study, where it was stated that completion of the DCDQ-ES was anonymous and voluntary. The e-mail address and telephone number of the first author were included in the letter so parents could contact the research team for clarification of the items or the questionnaire. Only parents who consented to participate returned the DCDQ-ES to the schools after completion within one week. Researchers retrieved the completed questionnaires from the schools.

### 2.2. Measurements

#### European-Spanish Version of the DCDQ (DCDQ-ES)

The DCDQ-ES is a 15-item parent questionnaire designed to screen motor coordination disorders in 5–15-year-old children [23]. Using a five-point Likert scale, parents are asked to evaluate how well their child performs certain motor daily activities compared with their peers (1 = *not at all like your child*; 2 = *a bit like your child*; 3 = *moderately like your child*; 4 = *quite a bit like your child*; 5 = *extremely like your child*). Items are divided into three subscales or factors: control during movement, fine motor/handwriting and general coordination. 

Total and subscale scores are calculated, where higher scores indicate better performance and the total score indicates whether a child has probable DCD with respect to three age groups (5–7 years 11 months; 8–9 years 11 months; and 10–15 years) [24]. The DCDQ usually takes about 10–15 min to complete [23], and it is a well-validated and recommended tool for assessing criterion B of the DSM-5 for a diagnosis of DCD [2,3].

The DCDQ was originally developed in English, and its original validation study using a large sample of Canadian children demonstrated good psychometric properties (Cronbach’s alpha = 0.94; sensitivity = 85%; specificity = 71%) [23]. 

Translation into European Spanish, cross-cultural adaptation and preliminary psychometric validation of the DCDQ-ES have been described in a previous study, demonstrating that it is conceptually and semantically equivalent to its English version and is a reliable measure for assessing motor coordination in Spanish children [34]. Additionally, the DCDQ-ES has a moderate and significant correlation with the Spanish version of the DCDDaily-Q (r = 0.406; ICC = 0.381; *p* < 0.001), which contributes to demonstrating its concurrent validity [33]. 

The DCDQ-ES is available in the Supplementary Materials (Table S1).

### 2.3. Statistical Analysis

Analyses were performed using SPSS version 24 (SPSS Inc., Chicago, IL, USA) and EQS 6.1 for Windows. To assess the goodness of fit, confirmatory factor analysis (CFA) was conducted using an unweighted least-squares estimation method (*n* = 540) [38,39,40]. A root-mean-square error of approximation (RMSEA) of < 0.08, a comparative fit index (CFI) of > 0.95 and a non-normed fit index (NNFI) of > 0.95 were indicators that the model fitted the data adequately [41,42].

Reliability of the DCDQ-ES was calculated using Cronbach’s alpha, with a value higher than 0.70 considered to be an indication of good internal consistency. Student’s *t*-test, analysis of variance (ANOVA) and Bonferroni post-hoc tests were used to determine the discriminant validity of the DCDQ-ES by calculating differences between the control group and the pDCD, pDCD only, pDCD/ADHD and pDCD/ASD groups for mean item scores and mean total and subscale scores. Discriminant validity of the DCDQ-ES across age groups was also tested using Student’s *t*-test.

Mean differences according to sex and age group were assessed with Student’s *t*-test and ANOVA analysis. Then, the 5th, 10th, 15th and 20th percentiles of the normative group were calculated for the DCDQ-ES total and subscale scores in the overall sample and within each of the three age groups. ROC computations were conducted and DCDQ-ES total score sensitivity, specificity and predictive values were calculated.

Finally, we explored the potential research consequences of adjusting DCDQ-ES scores for the Spanish population by examining the percentage of children identified as having probable DCD using the original Canadian cut-offs (≤ 46 for ages 6–7; ≤ 55 for ages 8–9; or ≤ 57 for ages 10–11) or the Spanish-adjusted 5th percentile cut-offs for each age group.

## 3. Results

### 3.1. Construct Validity and Internal Consistency

**Figure 1:**
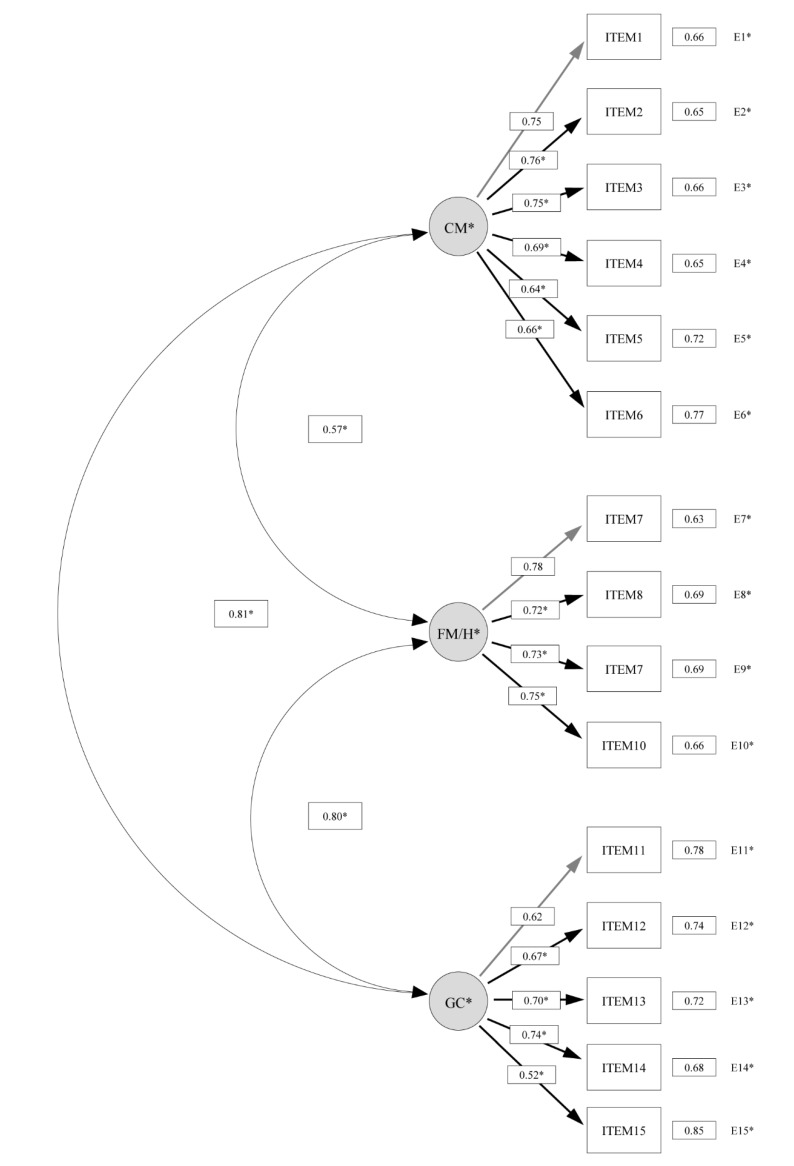
Confirmatory factor analysis of the Spanish version of the Developmental Coordination Disorder Questionnaire (DCDQ-ES) three-factor model (*n* = 540). Items 1, 7 and 11 fixed to 1 during estimation. CM = control during movement; FM/H = fine motor/handwriting; GC = general coordination.

The original proposed three-factor model reported an overall good fit to the data (factor 1 = control during movement *(items 1–6)*; factor 2 = fine motor/handwriting *(items 7–10)*; factor 3 = general coordination *(items 11–15)*) (X^2^(87) = 667.7, *p* < 0.01; CFI = 0.974; NNFI = 0.969; RMSEA = 0.047, 95% CI = 0.038–0.056). All the loadings were significant and ranged from 0.52 to 0.78 (Figure 1).

Internal consistency was excellent for the DCDQ-ES total, and good for the three motor coordination factors (DCDQ-ES total, α = 0.907; control during movement, α = 0.863; fine motor/handwriting, α = 0.835; general coordination, α = 0.775). The Cronbach’s alpha did not increase if any of the items were deleted, therefore indicating that no item was problematic.

### 3.2. Discriminant Validity

As displayed in Table 1, the total score of the DCDQ-ES showed a good discriminant capacity between typically developing children and children with probable DCD across age groups.

The pDCD group scored significantly lower than the matched control group, both for the DCDQ-ES total and subscale scores and all items. Children with pDCD only (without ADHD or ASD) also showed significantly poorer scores on the DCDQ-ES total scale and all subscales (Table 2). 

### 3.3. Age and Sex Differences and Age-Specific Cut-Offs

Significant differences between age groups were found in the DCDQ-ES total scale and all subscales (*p* < 0.001). Younger children scored significantly lower than their older peers in the DCDQ-ES total scale and subscales. 

Differences between sex groups were found only in one subscale. In the overall normative sample, girls scored significantly higher than boys in fine motor/handwriting (*p* < 0.001), but not in control during movement (*p* = 0.424), general coordination (*p* = 0.084) or total score (*p* = 0.228). 

**Figure 2:**
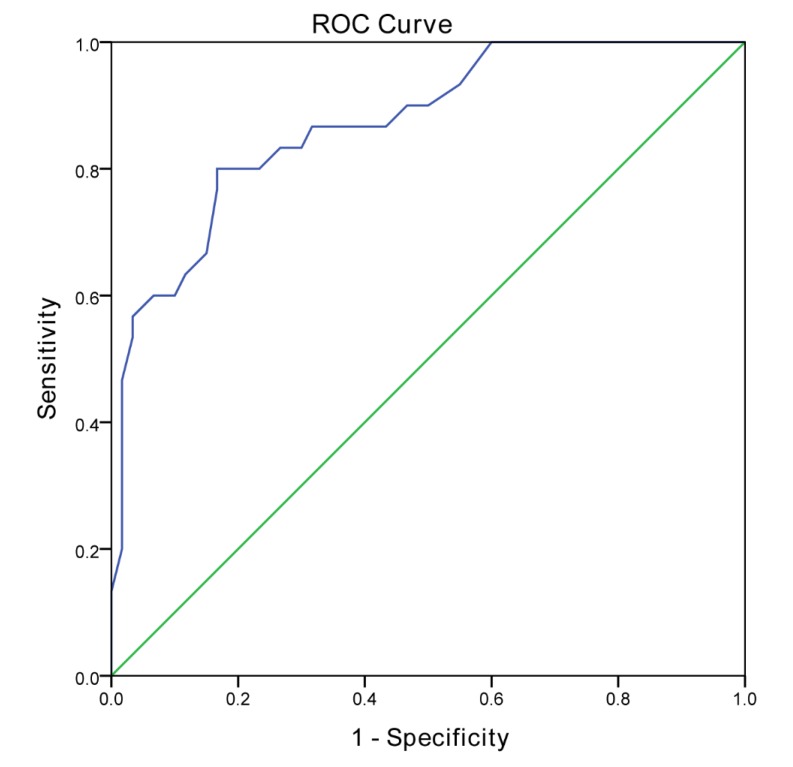
Receiver operating characteristic curve for the DCDQ-ES (cut-off score of 57) (*n* = 90).

Therefore, percentiles for all subscales and total score were calculated separately for each age group. In total, four cut-off points for each age group were calculated according to the 5th, 10th, 15th and 20th percentiles on the normative group for DCDQ-ES total and subscales (Table 3). The 15th percentile cut-off point of the DCDQ-ES for the total sample was 57 or below, with a sensitivity of 76.7% and a specificity of 83.3% (AUC = 0.872, 95% CI = 0.798 − 0.948, *n* = 90) (Table 4; Figure 2).

Table 5 displays the research consequences of using the original Canadian cut-off points for identifying Spanish children with probable DCD, which were developed using logistic regression modelling [23]. As observed, 3.5% of children in the reference sample were diagnosed differently, depending on the cut-off point used. For the youngest children there is a 100% rate of agreement between both cut-off proposals, but in older groups this mismatch would result in a high rate of false-positive diagnoses. This mismatch is especially relevant in children aged 10 to 11 years, as 6.7% of Spanish children would get a false positive of probable DCD in research practice.

## 4. Discussion

The aim of this research was to further validate the Spanish version of the DCDQ and to develop cut-off points for Spanish children using a randomly selected, sex and age-balanced sample of 540 Spanish typically developing children.

As motor coordination performance is a complex construct, different theories have been suggested and tested for its categorization when using and interpreting the DCDQ [23,43,44]. In this study, CFA analysis confirmed the original proposed three-factor structure, which is in line with the findings from Rivard et al. [44] and the validation study of the Italian version of the DCDQ [35]. Overall, these findings add to the evidence that motor coordination is a complex and multifactorial construct and that fine motor skills, coordination during movement and general coordination are interrelated factors but with unique differential aspects. For instance, girls and boys tend to show different motor coordination patterns in fine and gross motor skills, even when children come from different countries and cultural environments [21,45], and children with DCD struggle with different areas of motor coordination [3,13]. Therefore, it is necessary to assess each factor when exploring for DCD or coordination difficulties in daily living. Based upon the presented results, the authors recommend taking into account the specific problems in each of the three subscales in addition to interpreting the total score when using the DCDQ in a clinical context.

The DCDQ-ES has been previously cross-culturally adapted to the Spanish population, demonstrating that it is culturally and conceptually equivalent to the original DCDQ, and the preliminary validation study showed that the DCDQ-ES is a reliable tool for assessing motor performance in typically developing Spanish children [34]. In line with previous studies, findings from this further validation work report higher internal consistency values for the DCDQ-ES total scale and for the three subscales [24,25,26,27,28,29,30,31]. Cronbach’s alpha values in other validation studies in European, Asian and Latin American populations range from 0.89 to 0.96 [24,25,26,27,28,29,30,31], demonstrating that the DCDQ is a reliable tool for assessing motor coordination and probable DCD.

The DCDQ-ES showed a high capacity to discriminate between children with and without probable DCD. The pDCD group scored significantly lower on all of DCDQ-ES items, the total scale and each of the three subscales (*p* < 0.05). The total score of the DCDQ-ES significantly discriminated children in the pDCD group across the three age groups as well. The co-occurrence rate of other neurodevelopmental conditions within the pDCD group is in line with the high prevalence rates reported by previous research, particularly regarding ADHD and ASD [3,14,46,47,48]. Children with ADHD frequently present with motor coordination difficulties and DCD [3,49], and it has been questioned whether ADHD and DCD may pose as a unique disorder, but research demonstrates that they show differential motor, executive functioning and sensory processing characteristics and disparities in brain underpinnings, adding to the evidence of both disorders being commonly overlapping but different conditions [7,50,51]. 

Co-occurrence between DCD and ASD has been less explored, partially because assessment of motor coordination difficulties in children with ASD is reasonably more complex. However, the DSM-5 states that co-occurrence between both disorders is possible and research suggests that it may be quite frequent [2,3,52,53,54]. A recent study using a large sample of children with ASD (*n* > 11,000) estimates that prevalence of risk of DCD in this population is as high as 86.9% [55]. Even if ASD commonly overlaps with DCD, research supports that both are different disorders with unique physiological and functional characteristics and intervention requirements [56]. For instance, Caeyenberghs et al. [57] found that children with DCD only and ASD only showed disorder-specific neural alterations, while children with both DCD and ASD exhibited distinct topological patterns, concluding that co-occurring children have a unique neural signature. 

In this study, most of the items significantly discriminated children with pDCD only, pDCD/ADHD and pDCD/ASD, although some items (i.e., item 4, 5 or 15) did not discriminate typically developing children from pDCD only children, which can be partially explained by the small sample size in this subgroup. However, the total and subscale scores of the DCDQ-ES significantly discriminated children with pDCD only, pDCD/ADHD and pDCD/ASD, thus supporting the discriminant validity of the DCDQ-ES.

As expected, significant differences between age groups were found in both the DCDQ-ES total scale and all subscales. Older children scored significantly higher than younger children, which adds to the evidence that children improve their motor skills as they grow, as has been theorized previously by several authors, thus supporting the use of age-specific cut-off points [21,23,35,44,46].

Findings regarding sex differences in motor performance vary highly across cultural contexts and measures of assessment [20,33,45]. In this study, boys and girls showed a similar score on the DCDQ-ES total scale but had significant differences in the fine motor/handwriting subscale. 

Outcomes regarding differences in motor coordination between boys and girls are inconclusive and vary according to country and measure of assessment [58,59,60]. For instance, Rivard et al. [44] reported that Canadian typically developing and DCD girls scored better on the DCDQ total scale than typically developing and DCD boys, respectively, while Caravale et al. [35] found that Italian boys and girls showed similar scores on the Italian DCDQ. Using the DCDDaily-Q, Delgado-Lobete et al. [45] found that both Spanish and Dutch girls showed better performance in fine motor activities than Spanish and Dutch boys, but differences in total performance varied according to sex and country. 

These outcomes are in line with the findings from this study, and suggest that motor performance is probably influenced by cultural factors and daily activity participation. On the other side, typically developing boys are usually more proficient in gross motor skills than typically developing girls, while girls usually outperform boys in fine motor skills, but there is generally a higher proportion of males than females reported with DCD [21,33,45,59,60]. Thereby, it is possible that impairments in gross motor skills may be more evident than difficulties in fine motor performance, which could lead to girls with coordination motor struggles to go unnoticed. 

As age was significantly associated with DCDQ-ES subscales and total scores, different cut-off points were calculated following the original age categorization of the DCDQ [23]. The resulting Spanish cut-offs reflected the lower mean scores found in typically developing Spanish children in comparison with the Canadian children, except for younger children. Identifying DCD in young children may be more complicated than in older children because motor performance is more variant and coordination difficulties can be overturned [3,33]. 

Country-adjusted cut-off points have also been developed for other Southern American and European versions of the DCDQ, and these are usually lower than the original ones [35,61]. The established cut-off points for Brazilian children are significantly lower than both the Canadian and the Spanish norms, indicating lower overall scores in the DCDQ for Brazilian children [61]. While Italian adjusted cut-off points are almost similar to the Spanish norms in younger children, they differ significantly in the 8–9 and 10–12-years-old groups [35], which in the Spanish situation may reflect an increasing improvement in motor performance with age [21]. This situation may be due to different motor coordination standards between North America and South America or Southern Europe, which are consistent with the different prevalence rates of probable DCD among these populations [21,60]. Interestingly, differences between Italian and Spanish cut-off points further support that variances in motor coordination performance exist even between regions that may be perceived as similar.

The 5th percentile is often taken as the cut-off point in tools designed to identify the risk of DCD in research [32,35,62,63,64], and so it is the cut-off point recommended by the authors when using the DCDQ in Spain to operationalize criterion B of the diagnostic criteria for DCD diagnosis in research practice. Conversely, the use of the 15th percentile is recommended in clinical practice. However, as the aim of the DCDQ-ES is to identify as many children with probable DCD as possible, different percentile scores are given so that researchers and healthcare practitioners can compare a child’s performance in each of the three factors and the total scale in relation to the normative sample, thereby detecting those children with mild motor coordination difficulties in order to prompt strategies to prevent further consequences. An additional recommendation for clinicians would be to not only be alert to the total DCDQ-ES score but to notice whether the child scores lower than their peers in a particular area (i.e., control during movement, fine motor/handwriting or general coordination), as children with DCD present with a variety of motor coordination issues.

As expected, the Spanish recommended cut-off score for clinical practice in the overall sample, regardless of age group, is higher than the Canadian value (57 vs. 53). It is interesting to note that although this overall cut-off resulted in quite similar sensitivity values (Spanish = 77%; Canadian = 81%), the specificity in the Spanish version is significantly higher (Spanish = 83%; Canadian = 65%). 

However, sensitivity and specificity values for the clinical proposed Spanish cut-off were similar with that of the original DCDQ and other cross-cultural adaptations [23,25,26,27,30,35]. For instance, sensitivity and specificity of the German version of the DCDQ for a clinic sample was 72.7% and 95%, while these values decreased to 30% and 86.7% in a community sample [25]. The Italian-adjusted cut-off scores resulted in a sensitivity of 59% and a specificity of 65% for a community-based sample [35], but these values increased to 88% and 96% if using a clinical DCD sample [27]. The sensitivity and specificity for Brazilian children is 73% and 86.6%, respectively [26], and the European French values are similar as well (sensitivity = 85%, specificity = 81.6%) [30]. 

One important finding in this study was that using the non-country-adjusted cut-off points for Spanish children resulted in a significant mismatch and a high rate of false-positive diagnoses of probable DCD, especially in children older than 7 years. As previously discussed, the discrepancy between Canadian and Spanish norms could be explained by differences in motor coordination standards between regions, which have been reported in previous studies across European and American populations [35,45,60,62]. It may be possible that parents from different cultural and geographical backgrounds have distinct standards on rating their child’s motor performance in comparison to other children. 

These findings show that it is crucial to develop and promote the use of country-adjusted norms in order to prevent misleading outcomes in clinical and research practice. Possible clinical consequences of mistakenly identifying children with probable DCD include not only economic and resource costs but also the cost of putting families and children through unnecessary stress and potentially delaying a definite diagnosis. As the DCDQ-ES aims to operationalize criterion B of the DSM-5 diagnostic criteria for DCD, a diagnosis of definite DCD only should be made after a comprehensive multidisciplinary evaluation [3,33]. An occupational therapy evaluation of the impact of motor deficits on a child’s activities in daily living has specific relevance in the diagnosis of DCD (criterion B). Therefore, it is recommended to include pediatric occupational therapists in the multidisciplinary team.

Some limitations of this study should be addressed. One important limitation was that a definite diagnosis of DCD could not be established in the pDCD group. However, only children who scored at the most restrictive cut-off in the DCDDaily-Q were included in the pDCD group. Another limitation regarding the pDCD group is that most severe cases (i.e., children who had been referred for motor coordination difficulties in addition to another potential neurodevelopmental condition) were more likely to be recruited in this study, which may constitute a bias. A second limitation is that the sample size of the 10–11-years-old group in the pDCD group was very small. Additionally, our sample did not include children aged 12–15, therefore the norms for the older age group should be considered when assessing Spanish children older than 11 years. Finally, intra-rater reliability, test-retest reliability and concurrent validity with objective motor test batteries were not tested. Future research directions might include gathering data from children with a definite diagnosis of DCD in order to further test the sensibility and specificity of the proposed cut-off scores.

## 5. Conclusions

The present study has both research and clinical implications as it reports further information about the psychometric properties of the European Spanish version of the DCDQ and provides the reference norms for Spanish children. Findings show that the DCDQ-ES is a reliable and valid instrument for assessing motor coordination issues and for identifying children with probable DCD in Spanish context. Age-specific cut-off points adjusted to the Spanish population are provided for research and clinical purposes. The DCDQ-ES is a cost-effective, accessible and reliable measure for easy and quick assessment of motor coordination that may prompt further and comprehensive evaluation of potential DCD if needed. Health practitioners working in pediatric primary care or with children, such as occupational and physical therapists, can benefit from these findings and use the DCDQ-ES to operationalize criterion B of the diagnostic criteria for DCD.

## Figures and Tables

**Table 1:** DCDQ-ES total and subscale scores for pDCD and matched control group across age groups (*n* = 90).

DCDQ-ES	pDCD	Control	*p* Value
	Mean (SD)	Mean (SD)	
Total (*n* = 90)			
Control during movement	20.4 (6.2)	26.8 (3.3)	<0.001
Fine motor/ handwriting	12.0 (5.2)	17.2 (3.0)	<0.001
General coordination	15.7 (5.5)	21.4 (3.4)	<0.001
DCDQ-ES total	48.1 (14.0)	65.5 (8.3)	<0.001
6–7 years (*n* = 33)			
Control during movement	18.2 (7.3)	26.1 (3.1)	0.005
Fine motor/ handwriting	10.5 (5.8)	16.3 (2.6)	0.008
General coordination	14.2 (5.7)	21.1 (3.2)	0.002
DCDQ-ES total	42.8 (15.3)	63.5 (7.7)	<0.001
8–9 years (*n* = 48)			
Control during movement	21.4 (5.0)	27.2 (3.6)	<0.001
Fine motor/ handwriting	13.2 (4.7)	17.8 (3.2)	0.002
General coordination	16.9 (5.3)	21.5 (3.7)	<0.001
DCDQ-ES total	51.4 (12.9)	66.4 (8.8)	<0.001
10–11 years (*n* = 9).			
Control during movement	23.0 (7.8)	27.7 (2.3)	0.410
Fine motor/ handwriting	11.7 (5.5)	17.7 (2.7)	0.056
General coordination	15.0 (6.6)	22.0 (3.0)	0.055
DCDQ-ES total	49.7 (12.7)	67.3 (7.2)	0.029

SD = standard deviation; pDCD = probable Developmental Coordination Disorder.

**Table 2:** DCDQ-ES total, subscale and item scores for pDCD and matched control group (*n* = 90).

DCDQ-ES	pDCD	pDCD only	pDCD/ ADHD	pDCD/ ASD	Control	*p* Value within Groups
	Mean (SD) (*n* = 30)	Mean (SD) (*n* = 16)	Mean (SD) (*n* = 10)	Mean (SD) (*n* = 4)	Mean (SD) (*n* =60)	
Item 1	3.0 (1.3)	3.4 (1.2)	2.9 (1.2)	1.8 (1.5)	4.4 (0.8)	<0.001 ^a^; 0.002 ^b^; <0.001 ^c^; <0.001 ^d^
Item 2	2.8 (1.3)	3.1 (1.1)	2.8 (1.4)	1.8 (1.5)	4.5 (0.7)	<0.001 ^a^; <0.001 ^b^; <0.001 ^c^; <0.001 ^d^
Item 3	2.7 (1.4)	2.9 (1.1)	2.7 (1.7)	2.0 (2.0)	4.1 (1.0)	0.004 ^a^; 0.004 ^b^; 0.004 ^c^; <0.001 ^d^
Item 4	4.0 (1.2)	4.3 (0.9)	4.1 (1.4)	3.0 (1.6)	4.6 (0.7)	0.033 ^a^; 1.000 ^b^; 0.760 ^c^; 0.004 ^d^
Item 5	4.0 (1.2)	4.1 (1.0)	4.3 (1.3)	2.8 (1.5)	4.6 (0.6)	0.016 ^a^; 0.213 ^b^; 1.000 ^c^; <0.001 ^d^
Item 6	3.8 (1.2)	4.1 (1.0)	3.8 (1.4)	3.0 (1.4)	4.8 (0.5)	<0.001 ^a^; 0.012 ^b^; 0.003 ^c^; <0.001 ^d^
Item 7	3.0 (1.5)	3.5 (1.4)	2.3 (1.1)	3.0 (2.3)	4.3 (0.8)	<0.001 ^a^; 0.053 ^b^; <0.001 ^c^; 0.119 ^d^
Item 8	3.0 (1.6)	3.6 (1.7)	2.5 (1.4)	1.8 (1.0)	4.4 (0.9)	<0.001 ^a^; 0.082 ^b^; <0.001 ^c^; <0.001 ^d^
Item 9	3.0 (1.4)	3.6 (1.3)	2.3 (1.1)	2.5 (1.7)	4.2 (1.0)	<0.001 ^a^; 0.357 ^b^; <0.001 ^c^; 0.017 ^d^
Item 10	3.0 (1.4)	3.4 (1.3)	2.6 (1.2)	2.0 (1.4)	4.3 (0.9)	<0.001 ^a^; 0.029 ^b^; <0.001 ^c^; <0.001 ^d^
Item 11	3.5 (1.5)	3.8 (1.5)	3.8 (1.1)	1.8 (1.0)	4.6 (0.7)	0.001 ^a^; 0.011 ^b^; 0.087 ^c^; <0.001 ^d^
Item 12	3.4 (1.4)	4.0 (1.2)	3.2 (1.2)	1.5 (1.0)	4.4 (0.7)	<0.001 ^a^; 0.666 ^b^; 0.001 ^c^; <0.001 ^d^
Item 13	2.7 (1.4)	3.3 (1.4)	1.8 (0.9)	2.3 (1.3)	4.3 (1.0)	<0.001 ^a^; 0.014 ^b^; <0.001 ^c^; 0.003 ^d^
Item 14	3.3 (1.5)	3.8 (1.4)	3.1 (1.4)	1.8 (1.0)	4.3 (1.0)	0.002 ^a^; 0.793 ^b^; 0.014 ^c^; <0.001 ^d^
Item 15	2.8 (1.5)	3.5 (1.5)	1.8 (0.9)	2.8 (1.7)	3.9 (1.1)	0.001 ^a^; 1.000 ^b^; <0.001 ^c^; 0.388 ^d^
Control during movement	20.4 (6.2)	21.8 (4.4)	20.6 (6.8)	14.3 (8.8)	26.8 (3.3)	<0.001 ^a^; <0.001 ^b^; <0.001 ^c^; <0.001 ^d^
Fine motor/ handwriting	12.0 (5.2)	14.2 (4.9)	9.7 (4.2)	9.3 (5.6)	17.2 (3.0)	<0.001 ^a^; 0.025 ^b^; <0.001 ^c^; <0.001 ^d^
General coordination	15.7 (5.5)	18.4 (5.0)	13.7 (3.8)	10.0 (5.5)	21.4 (3.4)	<0.001 ^a^; 0.035 ^b^; <0.001 ^c^; <0.001 ^d^
DCDQ-ES total	48.1 (14.0)	54.3 (10.7)	44.0 (11.8)	33.5 (18.6)	65.5 (8.3)	<0.001 ^a^; <0.001 ^b^; <0.001 ^c^; <0.001 ^d^

SD = standard deviation; pDCD = probable Developmental Coordination Disorder; = ADHD = Attention Deficit and Hyperactivity Disorder; ASD = Autism Spectrum Disorder; ^a^ = between controls and pDCD; ^b^ = between controls and pDCD only; ^c^ = between controls and pDCD/ADHD; ^d^ = between controls and pDCD/ASD.

**Table 3:** Overall and age-specific cut-off points according to the 5th, 10th, 15th and 20th percentiles for the DCDQ-ES total and subscores in the normative group (*n* = 540).

DCDQ-ES Total and Subscales	p5	p10	p15	p20
Normative group (*n* = 540)				
Control during movement	19	21	22	24
Fine motor/ handwriting	12	14	15	16
General coordination	15	17	18	19
DCDQ-ES	**49**	55	**57**	59
6–7 years old				
Control during movement	18	20	21	22
Fine motor/ handwriting	12	13	14	15
General coordination	14	16	17	18
DCDQ-ES	**46**	50	**54**	57
8–9 years old				
Control during movement	19	21	22	24
Fine motor/ handwriting	12	14	15	16
General coordination	15	17	19	19
DCDQ-ES	**50**	55	**58**	60
10–12 years old				
Control during movement	21	23	24	24
Fine motor/ handwriting	13	15	16	16
General coordination	16	17	19	20
DCDQ-ES	**53**	56	**59**	61

In bold = recommended cut-offs for DCD indication (criterion B) in clinical practice (p15) and research (p5).

**Table 4:** Predictive values and Youden’s index of the 5th and the 15th percentiles (*n* = 90).

DCDQ-ES Total Score	FP N (%)	FN N (%)	TP N (%)	TN N (%)	PPV	NPV	Youden’s Index
p15 (57)	10 (16.7)	7 (23.3)	23 (76.7)	50 (83.3)	69.7%	87.7%	0.833
p5 (49)	1 (1.7)	16 (53.3)	14 (46.7)	59 (98.3)	93.3%	78.7%	0.450

FP = false positive; FN = false negative; TP = true positive; TN = true negative; PPV = positive predictive value; NPV = negative predictive value.

**Table 5:** Prevalence of children diagnosed with probable DCD using Canadian or Spanish cut-off points (*n* = 540).

	Canadian Cut-Offs	
Spanish Cut-Offs	Probable not DCD	Probable DCD
Total sample		
Probably not DCD	90.9%	3.5%
Probable DCD	0.0%	5.6%
6–7 years old		
Probably not DCD	95.0%	0.0%
Probable DCD	0.0%	5.0%
8–9 years old		
Probably not DCD	90.5%	3.9%
Probable DCD	0.0%	5.6%
10–12 years old		
Probably not DCD	87.2%	6.7%
Probable DCD	0.0%	5.1%

DCD = Developmental Coordination Disorder.

## Supplementary Materials

The following are available online at https://www.mdpi.com/1660-4601/17/7/2425/s1, Table S1: European Spanish version of the DCDQ.

## Appendix A

**Table A1:** Age and sex for all groups (*n* = 570).

Participants	N	Boys (N (%))
Normative group	540	270 (50.0%)
6–7 years old	180	90 (50.0%)
8–9 years old	180	90 (50.0%)
10–12 years old	180	90 (50.0%)
pDCD group	30	20 (66.7)
6–7 years old	11	8 (72.7)
8–9 years old	16	10 (62.5)
10–11 years old	3	1 (33.3)
Control group	60	40 (66.7)
6–7 years old	32	16 (72.7)
8–9 years old	32	20 (62.5)
10–11 years old	6	2 (33.3)
