# Supplementary material for: Psychometric Validation and Reference Norms for the European Spanish Developmental Coordination Disorder Questionnaire: DCDQ-ES
Source: Int J Environ Res Public Health. 2020 Apr 2;17(7):2425. doi: 10.3390/ijerph17072425 (PMC7178027; doi:10.3390/ijerph17072425)
Supplement: Supplementary file 1 [file ijerph-17-02425-s001.pdf]

European Spanish DCDQ: DCDQ-ES

1 = Muy diferente a como lo hacen otros niños [*Not at all like your child*].
2 = Algo parecido a como lo hacen otros niños [*A bit like your child*].
3 = Moderadamente parecido a como lo hacen otros niños [*Moderately like your child*].
4 = Muy parecido a como lo hacen otros niños [*Quite a bit like your child*].
5 = Prácticamente igual a como lo hacen otros niños [*Extremely like your child*].

	1	2	3	4	5
Su hijo/a lanza una pelota de forma controlada y precisa [*Your child throws a ball in a controlled and accurate fashion]*					
Su hijo/a atrapa una pelota pequeña (p.e.: del tamaño de una pelota de tenis) lanzada desde una distancia de aproximadamente 2 metros [*Your child catches a small ball (e.g., tennis ball size) thrown from a distance of 6 to 8 feet (1.8 to 2.4 meters)*]					
Su hijo/a golpea de forma precisa con una raqueta una pelota pequeña en movimiento [*Your child hits an approaching ball or birdie with a bat or racquet accurately*]					
Su hijo/a salta sin dificultad los obstáculos que se pueden encontrar en el jardín o en una zona de juego [*Your child jumps easily over obstacles found in garden or play environment*]					
Su hijo/a corre de manera similar y tan rápido como otros niños/as de su misma edad y sexo [*Your child runs as fast and in a similar way to other children of the same gender and age*]					
Si su hijo/a se propone realizar una actividad motora, puede dirigir sus movimientos adecuadamente para llevar a cabo la tarea y completarla de forma efectiva (p.e.: al construir una "muralla" o "torre" con bloques o cojines, al manejar los juguetes, al construir una estructura con bloques, o al usar material de manualidades) [*If your child has a plan to do a motor activity, he/she can organize his/her body to follow the plan and effectively complete the task (e.g., building a cardboard or cushion "fort," moving on playground equipment, building a house or a structure with blocks, or using craft materials)*]					
En clase, su hijo/a copia, escribe o dibuja lo suficientemente rápido como para seguir el ritmo del resto de sus compañeros/as [*Your child's printing or writing or drawing in class is fast enough to keep up with the rest of the children in the class*]					
Su hijo/a copia o escribe números, letras o palabras de forma legible, precisa y correcta. Si su hijo/a aún no escribe, es capaz de colorear y dibujar de manera coordinada, y hace dibujos que se puedan reconocer [*Your child's printing or writing letters, numbers and words is legible, precise and accurate or, if your child is not yet printing, he or she colors and draws in a coordinated way and makes pictures that you can recognize*]					
Su hijo/a ejerce la fuerza o tensión correctas al escribir o al dibujar (es decir, no aplica excesiva presión en el trazo ni agarra con excesiva fuerza el lápiz, y su escritura no es ni demasiado fuerte ni demasiado suave sobre el papel) [*Your child uses appropriate effort or tension when printing or writing or drawing (no excessive pressure or tightness of grasp on the pencil, writing is not too heavy or dark, or too light)]*					

Su hijo/a recorta dibujos y formas fácilmente y de manera precisa<br>[*Your child cuts out pictures and shapes accurately and easily*]					
Su hijo/a muestra interés y le gusta participar en deportes o juegos activos que requieran buenas habilidades motoras<br>[*Your child is interested in and likes participating in sports or active games requiring good motor skills*]					
Su hijo/a aprende nuevas actividades motoras (p.e.: nadar, patinar) fácilmente y sin necesitar más práctica o tiempo que otros niños/as para alcanzar el mismo nivel de habilidad<br>[*Your child learns new motor tasks (e.g., swimming, rollerblading) easily and does not require more practice or time than other children to achieve the same level of skill*]					
Su hijo es rápido y eficaz al asearse o arreglarse, al ponerse y atarse los zapatos, al vestirse, etc.<br>[*Your child is quick and competent in tidying up, putting on shoes, tying shoes, dressing, etc.*]					
Su hijo/a se mueve y se desplaza sin torpeza, sin chocar con objetos ni romper cosas frágiles cuando se mueve en una habitación pequeña (es decir, no se le describiría como un "niño torpe" o como un "elefante en una cacharrería")<br>[*Your child would never be described as a "bull in a china shop" (that is, appears so clumsy that he or she might break fragile things in a small room)*]					
Su hijo/a es capaz de mantener la postura sin cansarse ni desplomarse o deslizarse por la silla, cuando se le pide que permanezca sentado durante cierto tiempo<br>[*Your child does not fatigue easily or appear to slouch and "fall out" of the chair if required to sit for long periods]*					
